# Prostate: simplified MRI anatomy recognition and training—the P-SMART study: a novel approach to defining prostatic apex, mid-gland and base

**DOI:** 10.1186/s13244-026-02459-2

**Published:** 2026-11-05

**Authors:** Samuel J. Withey, Patricia Gutierrez, Alfonso M. Ponsiglione, Arnaldo Stanzione, Jeries P. Zawaideh, Giorgio Brembilla, Giacomo Avesani, Charlie A. Hamm, Adriano B. Dias, Sungmin Woo, Anwar R. Padhani, Vibeke Løgager, Andrea Ponsiglione, P-SMART Collaborators, Jorge Abreu-Gomez, Georgios Agrotis, Francesco Alessandrino, Fahd Alkhalifah, Yuki Arita, Aishah Azam, Tristan Barrett, Mariem Bensalah-Faria, Rodrigo Borrero-Leon, Silvia Bottazzi, Donato Bressi, Iztok Caglic, Charlotte Charbel, Emil Colliander, Renato Cuocolo, Maarten De Rooij, Nando De Vulder, Margot Delrue, Sofia A. N. Fialho, Maria G. Freda, Matilde L. R. R. Gonçalves, Jonathan Hernández-Mancera, Ilias Karvounis, Iman Kiani, Fredrik Langkilde, Nicolas A. Litardo, Ashley Luckyram, Luigi Mannacio, Ricardo S. Martins, Sandra P. Mazzini, Ana Sofia L. Moreira, Shakiba Mosadegh, Jina Pakpoor, Pavel Radu, Giorgio Raia, Guillaume Ramette, Luca Russo, Daniel A. Sandoval, Alicia R. Silberbrandt, William Weston, Chen Jiang Wu, Lili Xu, Yu Hang Yang

**Affiliations:** 1 The Royal Marsden NHS Foundation Trust, London, UK; 2 Department of Imaging, Radboud UMC Research Institute of Medical Innovation, Radboud UMC, Nijmegen, Netherlands; 3 CHU Lille, Lille, France; 4 Department of Electrical Engineering and Information Technology, University of Naples ‘Federico II’, Naples, Italy; 5 Department of Advanced Biomedical Sciences, University of Naples “Federico II”, Naples, Italy; 6 Department of Radiology, IRCCS Ospedale Policlinico San Martino, Genova, Italy; 7 IRCCS San Raffaele Scientific Institute, Milan, Italy; 8 Fondazione Policlinico Universitario Agostino Gemelli, Rome, Italy; 9 Department of Radiology, Charité Universitätsmedizin Berlin, Berlin, Germany; 10 Berlin Institute of Health, Berlin, Germany; 11 University Medical Imaging Toronto, Joint Department of Medical Imaging, University Health Network, Mount Sinai Hospital and Women’s College Hospital, Department of Medical Imaging, University of Toronto, Toronto, ON Canada; 12 Department of Radiology, NYU Grossman School of Medicine, New York, NY USA; 13 Paul Strickland Scanner Centre, Mount Vernon Hospital, Northwood, UK; 14 Herlev-Gentofte Hospital, Copenhagen, Denmark; 15 The Netherlands Cancer Institute, Amsterdam, Netherlands; 16 University of Miami, Miami, FL USA; 17 University of California, San Francisco, CA USA; 18 Guy’s and St Thomas’ Hospital NHS Trust, London, UK; 19 Addenbrooke’s Hospital, University of Cambridge, Cambridge, UK; 20 Hôpital Fribourgeois, Villars-sur-Glâne, Switzerland; 21 University Medical Imaging Toronto—University of Toronto, Toronto, ON Canada; 22 Fondazione Policlinico Universitario A. Gemelli IRCCS, Rome, Italy; 23 Centre for Medical Imaging, University College London, London, UK; 24 Department of Radiology, University College London Hospital, London, UK; 25 NYU Langone Health, New York, NY USA; 26 University of Salerno, Salerno, Italy; 27 Radboud University Medical Center, Nijmegen, Netherlands; 28 University Hospital Ghent, Ghent, Belgium; 29 Hospital de Faro, Faro, Portugal; 30 Fundació Puigvert, Barcelona, Spain; 31 General University Hospital of Larissa, Mezourlo, Larissa, Greece; 32 Tehran University of Medical Sciences, Tehran, Iran; 33 University of Gothenburg, Göteborg, Sweden; 34 Jackson Memorial Hospital, Miami, FL USA; 35 IPO Francisco Gentil Porto, Porto, Portugal; 36 Hudiksvalls Sjukhus, Hudiksvall, Sweden; 37 Hôpital fribourgeois (HFR), Villars-sur-Glâne, Switzerland; 38 Centre Hospitalier de Valenciennes, Valenciennes, France; 39 Hospital Universitari de Bellvitge, Barcelona, Spain; 40 The First Affiliated Hospital with Nanjing Medical University, Nanjing, China; 41 Zhejiang Cancer Hospital, Hangzhou, China

**Keywords:** Education, Magnetic resonance imaging, Observer variation, Prostate, Prostate/anatomy and histology

## Abstract

**Objectives:**

Precise lesion localisation on prostate MRI—specifically, identification of whether at the apex, mid-gland, or base—is essential for effective communication and clinical management. This study aimed to assess interobserver agreement of current practice using the Prostate Imaging-Reporting and Data System (PI-RADS) against a novel anatomy-based approach, P-SMART (Prostate Simplified MRI Anatomy Recognition and Training).

**Materials and methods:**

This multi-reader, two-phase agreement study, used axial and sagittal T2-weighted MRI from a publicly available dataset. Forty MRI cases were annotated with four regions of interest (ROIs). Readers were asked to document ROI location—apex, mid-gland, or base—first using the PI-RADS method, then following a 3-week washout period and short educational intervention, using the P-SMART method. Interobserver agreement was evaluated using Fleiss’ kappa (κ) statistics and compared following bootstrap resampling using a paired *t*-test. Subgroup analyses were performed by reader expertise and prostate size.

**Results:**

Following quality assurance, 156 ROIs were evaluated by 43 readers in each phase of the study. Overall interobserver agreement improved with P-SMART vs PI-RADS (κ = 0.571 vs 0.399; *p* < 0.001). In subgroup analyses, improvements were consistent for experts (0.663 vs 0.435; *p* < 0.001), non-experts (0.530 vs 0.393; *p* < 0.001), large prostates (0.518 vs 0.325; *p* < 0.001), and small prostates (0.620 vs 0.461; *p* < 0.001).

**Conclusion:**

The P-SMART approach, designed to align with the PI-RADS sector map, yielded significantly higher interobserver agreement for prostate lesion-level localisation than the PI-RADS method, across reader experience and gland size, though overall agreement was only moderate. Improved reproducibility may support biopsy targeting, active surveillance monitoring, and treatment planning.

**Key Points:**

***Question*** Can a novel method of dividing the prostate into apex, mid-gland, and base improve reader agreement compared to current practice?

***Findings*** The P-SMART method to divide the prostate outperformed PI-RADS (Fleiss’ kappa 0.571 vs 0.399), improving consistency across experience levels and prostate sizes.

***Relevance statement*:**

More reproducible prostate level assignment supports accurate lesion localisation for biopsy planning, active surveillance, and treatment planning. If validated, P-SMART may improve communication and could be integrated into reporting workflows and software tools.

**Graphical Abstract:**

**Figure:**
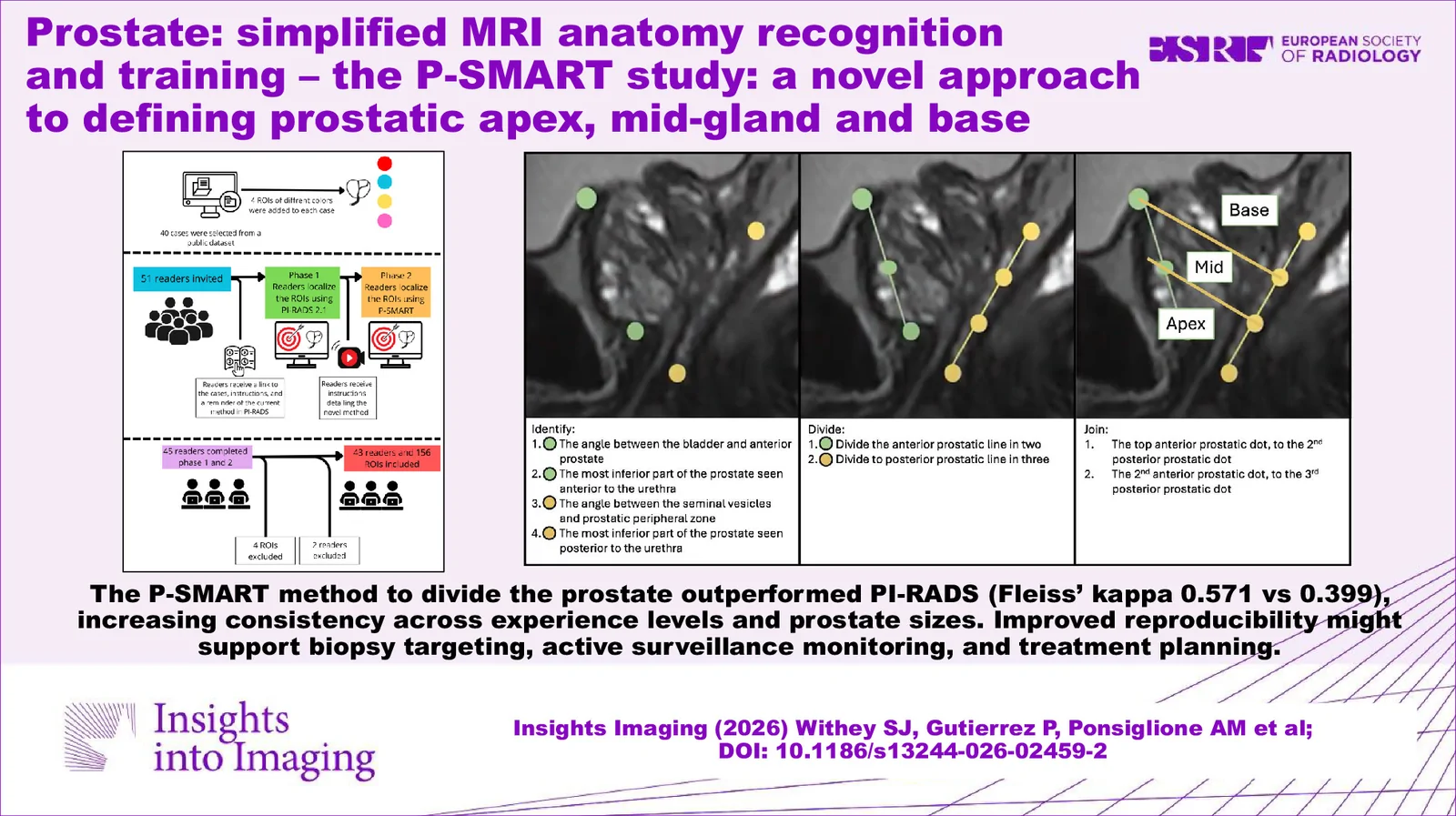

## Introduction

Magnetic resonance imaging (MRI) plays a central role in the prostate cancer (PCa) pathway, from diagnosis and screening, to staging, monitoring of disease, treatment planning, and detection of recurrence [1]. Scoring systems have been devised to standardise interpretation of prostate MRI, and to improve communication between the radiologist writing the report, and the physician reading it [2].

When reporting prostate MRI, it is important to document the location of lesions within the prostate gland. To aid this, the Prostate Imaging Reporting and Data System (PI-RADS) guidance includes a sector map that divides the prostate into 38 segments, plus one for each seminal vesicle and the external urethral sphincter. It splits the prostate into three levels: apex, mid-gland, and base [3].

**Fig. 1:**
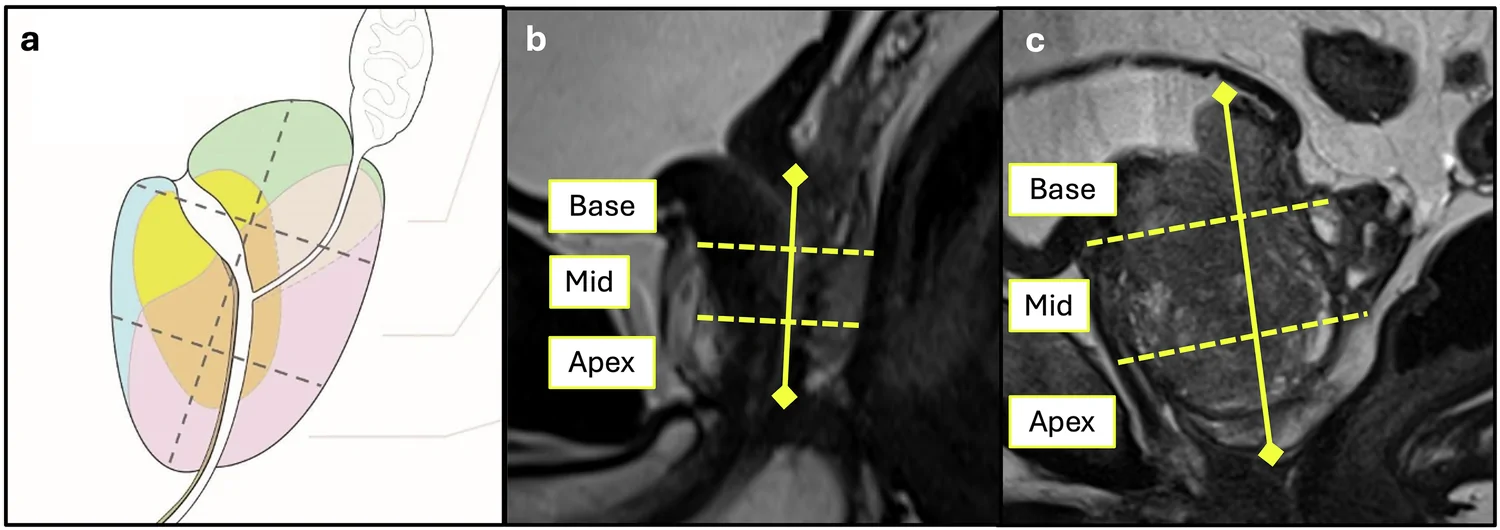
PI-RADS sector map, demonstrating how the prostate can be divided into three levels: apex, mid-gland, and base (**a**). Examples of how the map can be applied in clinical cases, in a ‘normal’ prostate (**b**), and in a patient with marked BPH (**c**). In **c**, strict adherence to the sector map results in the central zone and the most superior portion of the peripheral zone being classified as mid-gland. PI-RADS, prostate imaging reporting and data system. Part A adapted (with automatic permission under STM guidance) from Turkbey et al [3]

PI-RADS does not provide detailed instructions for this, although the sector map diagram appears to show the prostate’s long-axis measurement divided into three equal parts (Fig. 1a). Whilst this approach might work well in a patient with a ‘normal-shaped’ prostate, many patients undergoing prostate MRI have benign prostatic hyperplasia (BPH), with or without median lobe hypertrophy. In such cases, applying the PI-RADS guidance could result in the most superior portion of the peripheral zone being classified as mid-gland level (Fig. 1b, c). At radical prostatectomy, whole mount specimens are sectioned perpendicular to the posterior surface of the prostate as per ISUP guidelines; the PI-RADS method of dividing the gland into thirds along its long axis may not produce divisions consistent with this [4].

Previous work has shown that when faced with a lesion, which could involve more than one level within the prostate, pairs of readers only agreed on the exact levels involved in 61.4% of cases [5]. This might be compounded as PI-RADS permits different axial orientations, which might contribute to uncertainty around prostatic level [6]. To date, formal kappa-based assessment of interobserver agreement specifically for prostatic level localisation has not been reported. A lack of agreement on which level of the prostate contains a lesion may have clinical consequences, including in biopsy planning, sequential monitoring of lesions in active surveillance, and in deciding on surgical approach, particularly when considering a nerve-sparing prostatectomy.

This study proposes a new method for dividing the prostate levels and compares its inter-observer agreement with that of the current PI-RADS-based approach.

## Material and methods

**Fig. 2:**
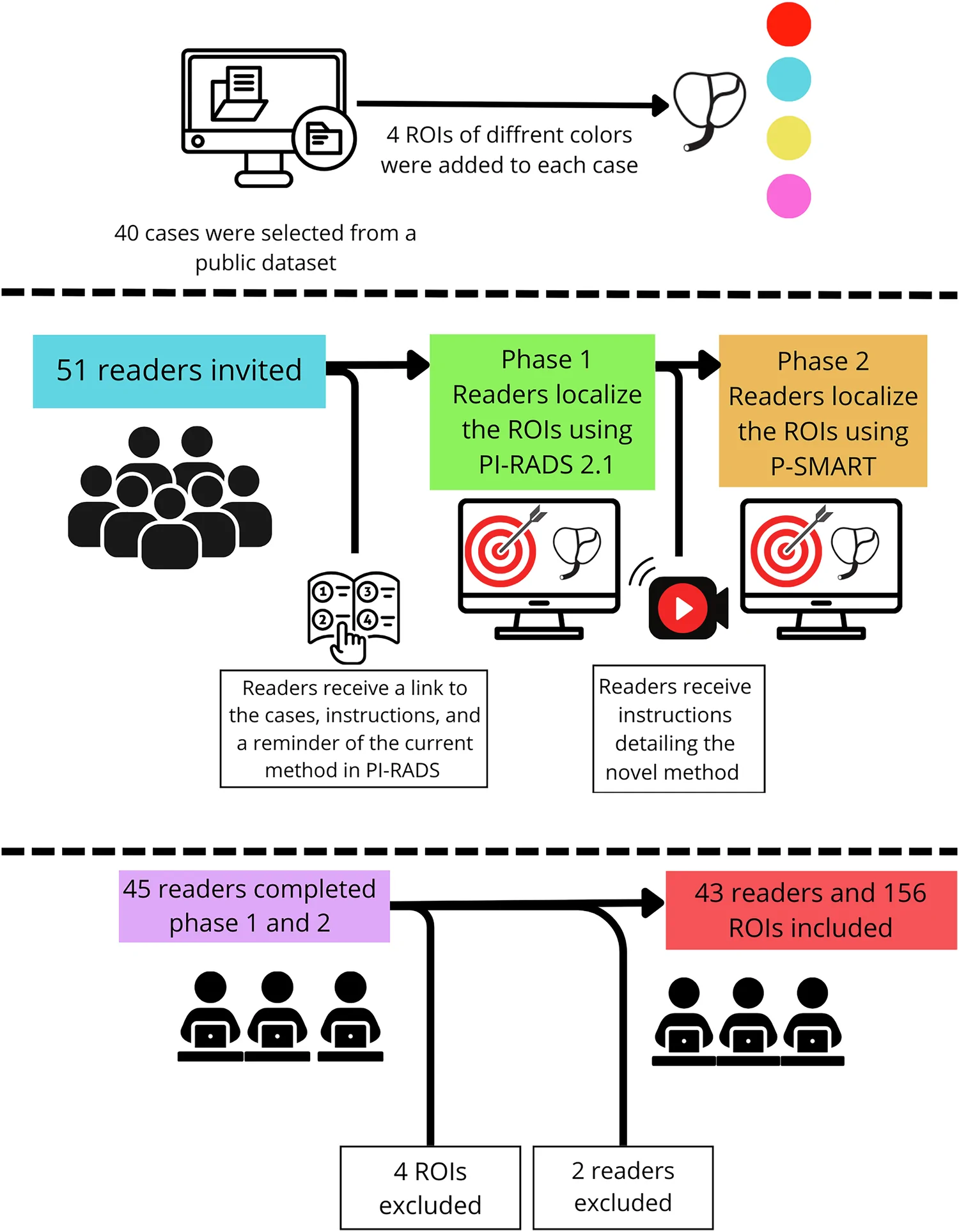
Schematic overview of the study

Institutional Review Board approval was not required, as all MRI cases were obtained from a publicly available dataset. The study outline is shown in Fig. 2.

### Prostate simplified MRI anatomy recognition and training (P-SMART) method

**Fig. 3:**
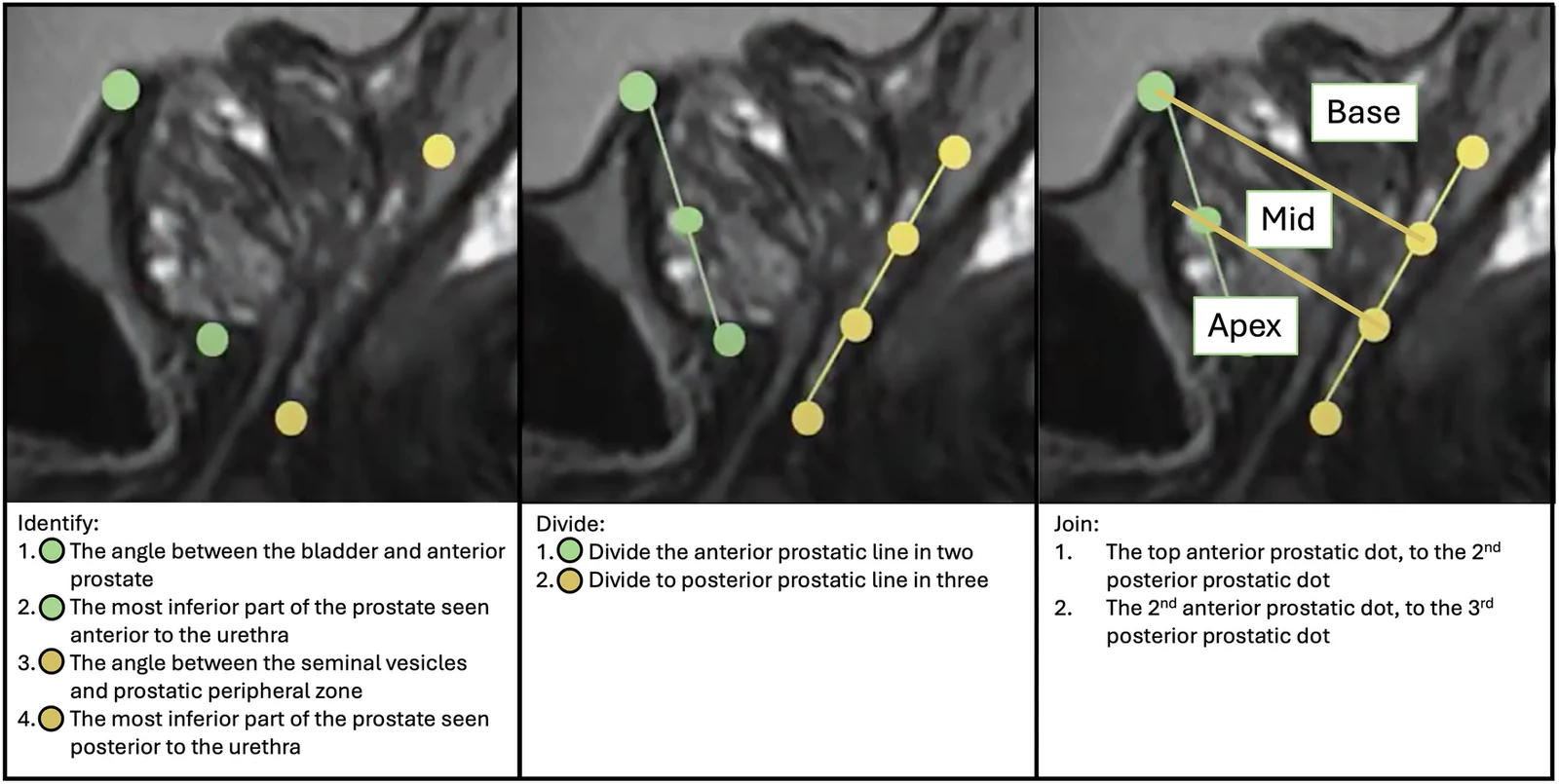
The P-SMART method demonstrated on a sagittal T2-weighted MRI. Three steps: Identify—four landmarks (the bladder-prostate angle anteriorly; the prostatic apex anterior to the urethra; the seminal vesicle-prostate angle posteriorly; the prostatic apex posterior to the urethra). Divide—the anterior line bisected; the posterior line trisected. Join—upper anterior midpoint to second posterior point (base/mid-gland boundary); lower anterior midpoint to third posterior point (mid-gland/apex boundary)

The novel method for dividing the prostate, illustrated in Fig. 3, relies on anatomical landmarks—specifically, the angles between the bladder and prostate, the seminal vesicles and prostate, and the most inferior portions of the prostatic apex anterior and posterior to the urethra—to provide a more reproducible delineation of the apex, mid-gland, and base.

### MRI dataset

Prostate MRI cases were downloaded from the publicly available ProstateX dataset (available from: https://prostatex.grand-challenge.org). 40 cases were selected to include a mixture of prostate morphologies, including varying degrees of BPH and median lobe hypertrophy, rather than by consecutive or random sampling. Prostate volumes were calculated using the ellipsoid formula as per PI-RADS v2.1, and ranged from 23 to 198 mL (median 60 mL; IQR 36–87 mL) [3]. In this dataset, the axial images were obliqued perpendicular to the path of the urethra.

The studies were acquired between 2011 and 2012 on Siemens 3-T scanners without use of an endorectal coil. T2-weighted images were acquired with a slice thickness of 3.6 mm. Sagittal and axial T2-weighted images were retained. Using Horos open-source PACS, four regions of interest (ROI) were added to all MRI studies, each represented by a 4mm-diameter circle of a distinct colour. The centre of each ROI was matched between axial and sagittal planes using the linking function. The cases were added to PACSbin, a web-based PACS (available at: http://www.pacsbin.com/).

### Reader identification and study workflow

Twenty prostate MRI radiologists from the European Society of Urogenital Radiology (ESUR) were invited to participate in the study and to involve less experienced colleagues, including residents with a demonstrated interest in prostate MRI. Those who accepted the invitation were sent instructions for Phase 1 of the study, which included a link to the PACSbin cases, directions on how to use the software, and a reminder of the definitions of apex, mid-gland, and base stated in PI-RADS [3]. Responses, including demographics, were collected using Google Forms (Alphabet). Phase 1 lasted three weeks, followed by a three-week washout period. The order of cases on PACSbin was rearranged for Phase 2. Readers who had completed Phase 1 of the study were invited to Phase 2.

Instructions were sent detailing the novel method for dividing the prostate with diagrams (Fig. 3) and a link to an 8-min instructional video (available at: https://www.youtube.com/watch?v=sRw1G6gqbb8). Again, data were collected using Google Forms (Alphabet) over 3 weeks.

### Statistical methods

The level of agreement between readers was assessed using Fleiss’ kappa coefficient (κ) [7]. The outcomes range from 1 (perfect agreement) to -1 (perfect disagreement), with 0 representing equal to chance. Fleiss kappa was estimated across all samples for the whole dataset, for both methods of dividing the prostate (PI-RADS & P-SMART). Subgroup analyses were performed based on reader experience level (> 1000 MRI studies independently read, classified as expert [8]), and prostate gland size (above vs below the dataset median volume).

To statistically compare the two rating approaches, a bootstrap resampling carried out over 1000 iterations was applied to estimate Fleiss’ Kappa distributions, and a paired *t*-test (Confidence = 95%; significance level α = 0.05) was carried out on the bootstrap distributions of Fleiss’ Kappa values. Descriptive statistics (mean; 95% confidence interval (CI)) for each Kappa distribution and the obtained *p*-values from statistical comparisons were computed. All statistical analyses were conducted using Python (v3.10) and standard scientific libraries.

## Results

Fifty-one potential readers agreed to take part in the study, 45 of whom completed both phases. The 45 Readers were from 16 countries. Two readers were excluded following a post-hoc quality assurance review, having consistently misidentified the apex and base across three quality assurance ROIs where all remaining readers showed unanimous agreement. This was done before any data analysis. Both readers would be classified as novices under ESUR/ESUI consensus criteria based on self-reported prior experience [8]. The final dataset of 43 Readers included 15 who were considered experts [8]. Of 160 ROIs, 4 had to be excluded due to mismatch between ROI location on axial and sagittal images, leaving a final dataset of 156 ROIs. The median prostate volume in the dataset was 60 mL. For subgroup analyses, glands with volumes > 60 mL were categorised as “large”, whereas those ≤ 60 mL were classified as “small”.

**Fig. 4:**
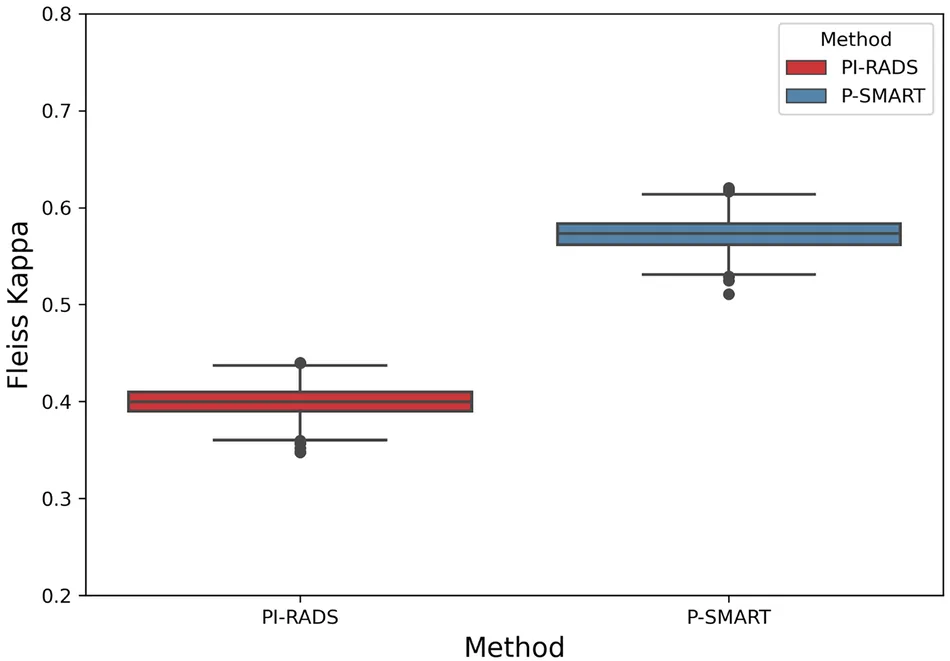
Overall consistency of readers’ responses as measured by Fleiss’ Kappa coefficient across the two methods (PI-RADS vs P-SMART). Bootstrap resampling (*n* = 1000 iterations) was applied to compute Fleiss’ Kappa distributions. PI-RADS, prostate imaging reporting and data system; P-SMART, prostate simplified MRI anatomy recognition and training

The distribution of inter-reader consistency values, as measured by Fleiss’ Kappa coefficient, for both methods of dividing the prostate (PI-RADS and P-SMART) is detailed in Table 1. For the PI-RADS-based method, interobserver agreement across all readers was 0.399, classified as “fair”. Using the P-SMART method led to a significant overall improvement, with κ increasing to 0.571, reflecting “moderate” agreement (*p* < 0.001) (Table 1 and Fig. 4).

**Table 1:** Descriptive statistics (mean, 95% CI) and *p*-values of paired *t*-test to compare Fleiss’ Kappa coefficient distributions across the two methods of dividing the prostate (PI-RADS vs P-SMART)

Group	Categories	Method	Mean Kappa	95% CI	*p*-value
Overall		P-SMART	0.571	0.542–0.603	< 0.001
		PI-RADS	0.399	0.368–0.427	
Prostate size	< 60 mL	P-SMART	0.620	0.571–0.666	< 0.001
		PI-RADS	0.461	0.416–0.505	
	≥ 60 mL	P-SMART	0.518	0.477–0.556	< 0.001
		PI-RADS	0.325	0.284–0.360	
Expertise level	Expert	P-SMART	0.663	0.630–0.700	< 0.001
		PI-RADS	0.435	0.401–0.468	
	Non-expert	P-SMART	0.530	0.497–0.562	< 0.001
		PI-RADS	0.393	0.362–0.423	

*P-SMART* prostate simplified MRI anatomy recognition and training, *PI-RADS* prostate imaging reporting and data system

**Fig. 5:**
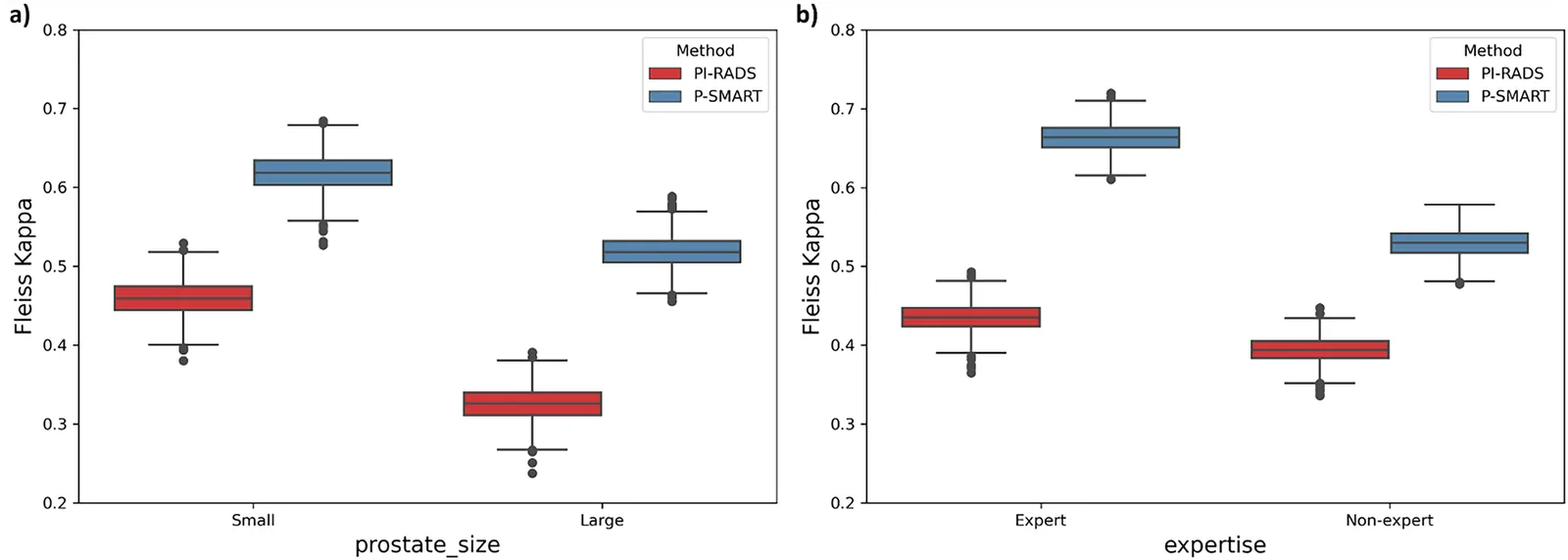
Consistency of readers’ responses as measured by Fleiss’ Kappa coefficient across the two methods (PI-RADS vs P-SMART). Readers’ responses were grouped: prostate size (large vs small) (**a**); expertise (**b**). Bootstrap resampling (*n* = 1000 iterations) was applied to compute Fleiss’ Kappa distributions. PI-RADS, prostate imaging reporting and data system; P-SMART, prostate simplified MRI anatomy recognition and training

Interobserver agreement was higher for P-SMART than PI-RADS when accounting for reader expertise and gland size: experts (κ = 0.663 vs 0.435, *p* = < 0.001), non-experts (κ = 0.530 vs 0.393, *p* = < 0.001), large prostates (κ = 0.518 vs 0.325, *p* = < 0.001) and small prostates (κ = 0.620 vs 0.461, *p* = < 0.001) (Table 1 and Fig. 5).

## Discussion

**Fig. 6:**
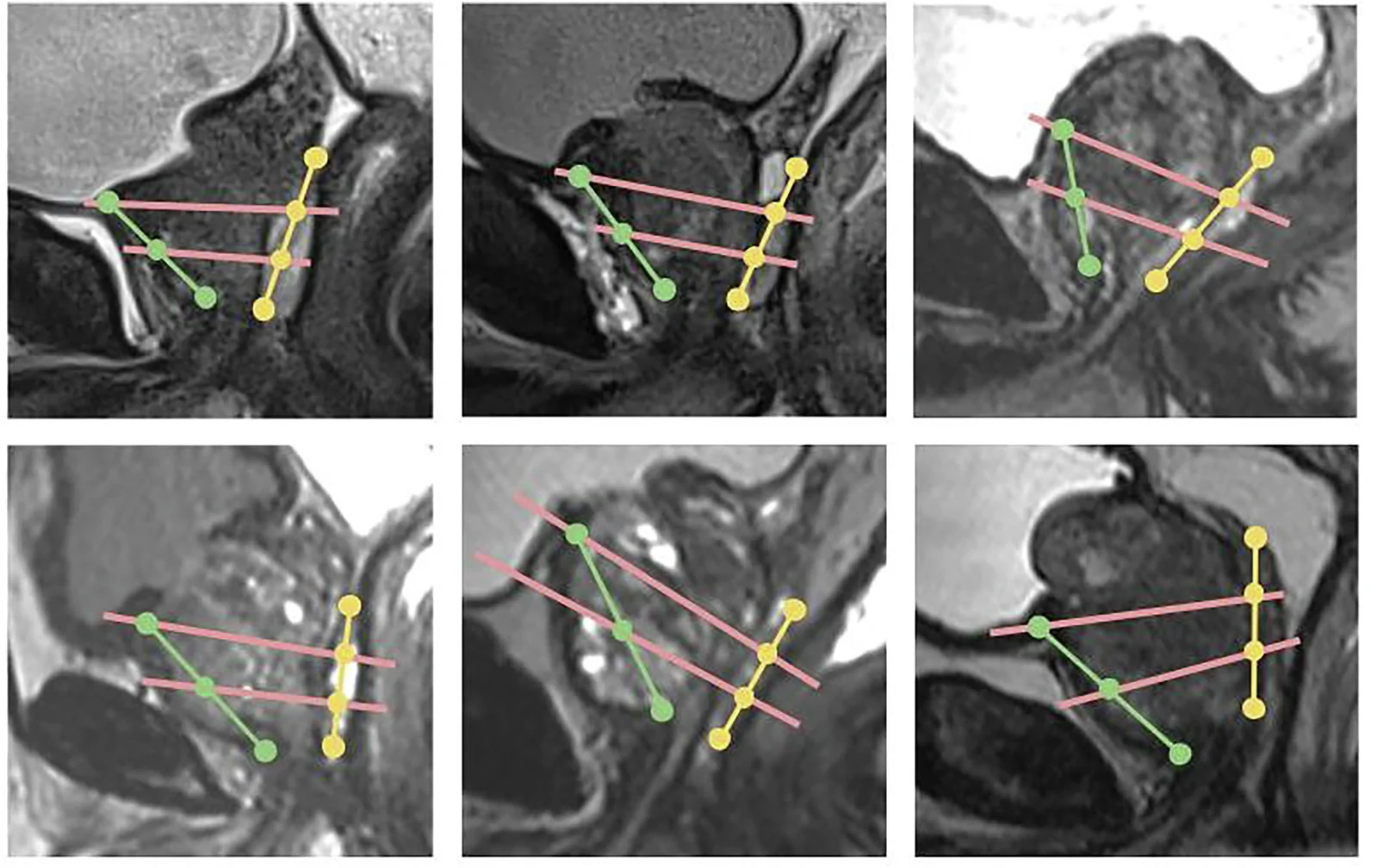
Six example cases, illustrating prostate segmentation using the P-SMART method. P-SMART, prostate simplified MRI anatomy recognition and training

Inspired by the 2021 critical review of PI-RADS 2.1, which highlights different interpretations of the long axis of the prostate [9], we devised a novel method of dividing the prostate into apex, mid-gland and base. As previously noted, the traditional division relies on convention, rather than well-defined anatomical landmarks. Therefore, we hypothesised that a more systematic, anatomy-based approach could improve agreement between readers [10]. The P-SMART method adapts to normal variation in prostate morphology, including those at the prostatic apex, and ensures that some peripheral zone is present at the base, even in cases of median lobe hypertrophy [11]. It does mean that in terms of relative volume, the base might be larger than the apex or mid-gland (Fig. 6), although there is no inherent issue with this.

In their study, Greer et al showed that, although inter-reader agreement for detecting index lesions on screenshots was satisfactory, introducing the PI-RADS sector map caused a dramatic drop in concordance [5]. Even with generous overlap of lesion outlines, agreement on exact sectors was only 21%, revealing substantial variability in how readers interpret the sector map. Previous work has shown that cognition-guided biopsies can be as good as image fusion, but inconsistency in lesion description poses a risk of miscommunication and inaccurate targeting during cognition-guided procedures [12]. Our findings are consistent with these results, as we observed only “fair” overall inter-reader agreement for ROI localisation using the conventional approach.

Following a short educational intervention, the novel P-SMART method demonstrated significantly improved interobserver agreement overall and in subgroup analyses stratified by reader expertise and prostate gland size. In this study, we selected interobserver agreement as the primary outcome measure to evaluate each method, rather than relying on comparison to a single expert reader. This better captures consistency across multiple observers and the reproducibility of each approach.

Interestingly, agreement of both methods was lower in large prostates compared to small ones. This was expected when using the PI-RADS method because BPH can cause the transition zone to extend much more cranially than the peripheral zone, which can lead to uncertainty. We speculate that with the P-SMART method, the anatomical landmarks could become more difficult to identify in larger glands.

The P-SMART method improved inter-observer agreement for both experts and non-experts. Even when using the novel method, which is designed to be more reproducible by being based on anatomical landmarks, experts still outperformed less experienced readers. This likely reflects the experts’ greater ability to accurately identify the anatomical landmarks required for precise prostate segmentation.

In the active surveillance setting for PCa, there is increasing and robust evidence supporting the use of MRI, with major guidelines advocating this technique for both patient selection and ongoing monitoring [13]. Accurate and reproducible MRI localisation of a known index lesion enables more precise monitoring of disease, including changes in lesion volume or signal characteristics, or the appearance of new lesions, thereby minimising the risk of disease misclassification. In the context of radiotherapy planning, precise mapping of lesions can optimise dose delivery while sparing adjacent healthy tissue, reducing potential toxicity [14]. Similarly, in surgical decision-making, knowledge of the exact location and extent is vital when planning nerve-sparing procedures, as mislocalization could compromise oncological control or functional outcomes [15].

The P-SMART method of dividing the prostate does have some limitations:

Small ROIs were used rather than true lesions, and readers were asked to document the single level that best represented the location of the ROI. However, in clinical practice, prostate lesions are often larger and may extend across multiple levels, which could influence localisation accuracy and inter-reader agreement. The use of artificial ROIs, whilst methodologically appropriate for isolating level assignment as the primary variable, means that performance with real lesions of varying size and conspicuity remains to be established.

We prioritised reproducibility over accuracy in this study. The goal was to assess how consistently readers applied PI-RADS and P-SMART rather than whether their interpretations were anatomically correct; i.e. there was no assessment against a proper “gold standard” as no anatomical reference standard for these boundaries exists. The classification of apex, mid-gland, and base represents a communication convention rather than a strictly defined anatomical truth; therefore, interobserver agreement constitutes the most clinically meaningful benchmark for these labels. This method only aims to improve agreement at the lesion level, whereas the PI-RADS sector map covers cross-sectional location in addition. It should also be noted that recruitment via ESUR may have introduced selection bias, as participants are likely to have greater familiarity with prostate MRI than the broader radiology community; consequently, performance in a general radiologist population may differ. Although less experienced colleagues were also included, these individuals were recruited through ESUR-affiliated participants, which may still reflect a relatively specialised cohort.

In addition, the exclusion of two readers based on quality assurance criteria was a post hoc decision made prior to formal data analysis and may represent an additional source of selection bias. These readers consistently inverted the prostate apex and base across ROIs with otherwise unanimous agreement, indicating a fundamental orientation error rather than variability in applying either method. Their inclusion would therefore not have provided meaningful information regarding the comparative performance of the evaluated approaches. Furthermore, this study included only radiologists. Whether P-SMART performs similarly among other professionals who routinely interpret prostate MR images and reports, such as urologists and radiation oncologists, remains unknown.

There is risk of instructional bias. All readers received written guidance for both phases: PI-RADS definitions for Phase 1; step-by-step instructions for the P-SMART method in Phase 2. In addition, an educational video was provided for the P-SMART method, whereas no video was given for the PI-RADS-based approach. Providing a video for current practice was deemed unnecessary, as PI-RADS relies on individual interpretation and does not offer a standardised, step-by-step protocol. However, this asymmetry reflects current clinical practice, in which PI-RADS relies on individual interpretation and does not prescribe a standardised, step-by-step protocol. As such, the difference in training between phases is inherent to the study design, which compares routine practice with a novel structured method. Although a formal statistical test for carryover effects was not performed, recall bias is unlikely to have materially influenced the results. The two methods are designed to yield different regional classifications, MRI cases were re-ordered between phases, and readers were required to localise 160 ROIs across 40 cases—a workload that limits precise recall over a three-week interval. More broadly, general learning effects, including increased familiarity with the study platform and refinement of assessment strategy over time, cannot be fully excluded as contributors to the observed improvement.

The novel approach relies on acquisition of sagittal T2-weighted imaging, which is not mandated by PI-RADS 2.1 [3]. The axial images within the dataset were obliqued perpendicular to the course of the prostatic urethra, therefore, the performance on MRI using true axial images has not been tested. However, as the sagittal sequence is the dominant sequence for dividing the prostate using the P-SMART method, performance is expected to be similar. Four of the original 160 ROIs were excluded due to technical mismatches between axial and sagittal planes, identified prospectively by readers during Phase 1 of the study and confirmed before any data analysis was performed; the same exclusion was applied to both phases. This highlights that the method is sensitive to precise image registration, and any errors in aligning ROIs (or in clinical practice, lesions) across planes could limit its clinical application. Images were acquired in 2011–2012 and may not reflect the image quality achievable with contemporary MRI hardware and acquisition protocols; performance of the P-SMART method on modern datasets warrants further evaluation.

It should be acknowledged that, despite significant improvement over current practice, agreement on ROI location was not perfect. Whilst P-SMART is “anatomy-based,” agreement was lower in cases with large prostates, and for non-expert readers. Identifying landmarks seems harder in these scenarios, indicating that even a structured approach still involves some subjective interpretation depending on patient anatomy. Variability in identifying the seminal vesicle–prostate junction, which may be slightly lateralized relative to the sagittal midline, could contribute to residual interobserver disagreement and represents an area for refinement. Additionally, prostate volumes in this historic dataset may not be representative of patients currently undergoing prostate MRI, given evolving referral pathways and trends towards earlier diagnosis.

Finally, although not quantitatively assessed in this study, the P-SMART method can be time-consuming, requiring approximately 1 min per case.

Future work might identify whether lesion localisation remains consistent under variations in bladder filling or rectal gas. Prospective clinical validation will ultimately be required to determine whether improved interobserver agreement with P-SMART translates into measurable gains in biopsy targeting accuracy, surgical planning, or radiation treatment field definition. As artificial intelligence solutions for automatic gland volume calculation and lesion identification become increasingly integrated in clinical practice [16], it should be feasible for developers to incorporate the P-SMART method into their workflows.

## Conclusion

In conclusion, the P-SMART approach for dividing the prostate yielded significantly higher interobserver agreement compared to current practice, regardless of reader experience or prostate gland size. This improvement highlights the method’s potential to standardise lesion localisation and reduce reporting variability, though overall agreement remained moderate, and prospective clinical validation is required before broader implementation.

## Data Availability

The annotated MRI dataset can be accessed at: https://www.pacsbin.com/collection/W1JKuTtSOj/review.

## Abbreviations

BPH: Benign prostatic hyperplasia
ESUR: European Society of Urogenital Radiology
PCa: Prostate cancer
PI-RADS: Prostate imaging reporting and data system
P-SMART: Prostate simplified MRI anatomy recognition and training
ROI: Regions of interest
